# Nosocomial Infections with IMP-19−Producing *Pseudomonas aeruginosa* Linked to Contaminated Sinks, France

**DOI:** 10.3201/eid2302.160649

**Published:** 2017-02

**Authors:** Lucie Amoureux, Karena Riedweg, Angélique Chapuis, Julien Bador, Eliane Siebor, André Péchinot, Marie-Lorraine Chrétien, Claire de Curraize, Catherine Neuwirth

**Affiliations:** University Hospital François Mitterrand, Dijon, France

**Keywords:** nosocomial infections, IMP-19, metallo-β-lactamases, *Pseudomonas aeruginosa*, bacteria, integrons, antimicrobial resistance, contaminated sinks, hospital, France

## Abstract

We isolated IMP-19–producing *Pseudomonas aeruginosa* from 7 patients with nosocomial infections linked to contaminated sinks in France. We showed that *bla*_IMP-19_ was located on various class 1 integrons among 8 species of gram-negative bacilli detected in sinks: *P. aeruginosa, Achromobacter xylosoxidans, A. aegrifaciens, P. putida, Stenotrophomonas maltophilia, P. mendocina, Comamonas testosteroni*, and *Sphingomonas* sp.

Acquired metallo-β-lactamases (MBLs) belong to the families IMP, VIM, NDM, SPM, GIM, SIM, DIM, KHM, TMB, FIM, and AIM (*1*). IMP and VIM are the most common families. MBLs have been reported worldwide among *Pseudomonas aeruginosa* isolates (*2*). Therapeutic options for infected patients are severely limited because isolates are resistant to many classes of antimicrobial drugs. Genes for MBLs are found mostly in class 1 integrons, which carry additional drug resistance genes. To date, 33 of the 51 known IMP variants have been detected in *P. aeruginosa*; there has been only 1 report of an IMP-19 producer (*3*). This MBL is widespread among *Acinetobacter* spp. in Japan and has also been reported in *Achromobacter xylosoxidans* (*4**,**5*).

During 2009–2016, infections with IMP-19–producing *P. aeruginosa* isolates were detected in 7 patients in the Hematology Department of University Hospital François Mitterrand, a 1,600-bed hospital in Dijon, France. We describe these infections and report results of environmental investigations.

## The Study

The hematology department of the hospital contains a 15-bed conventional unit and a 9-bed protective isolation unit. At the entrances of rooms in the conventional unit, there is a hand hygiene sink (for staff and visitors) and a bathroom in a separate area (shower stall, sink, and toilets). In the protective isolation unit, air is filtered through a laminar flow system, and a sink and toilets are located next to each bed (distance 1.5 m). The ceramic sinks have no counter space, and drains are made of stainless steel. All faucets in the department are hand-operated and provided with antibacterial filters (0.22 μm). Surfaces are cleaned daily (once in the conventional unit) and twice in the protective isolation unit with a solution containing quaternary ammonium compounds (0.25% didecyldimethylammonium chloride).

During 2009–2016, a total of 7 patients (P1–P7) in the department were infected or colonized by IMP-19–producing *P. aeruginosa*, which were isolated from blood samples (2 patients), urine samples (2 patients), throat swab samples (2 patients), and a central venous catheter (1 patient). All patients underwent throat, urine, and feces sampling at admission and were free of *P. aeruginosa,* thus indicating nosocomial acquisition. All isolates were resistant to ceftazidime, imipenem, meropenem, doripenem, ciprofloxacin, and most aminoglycosides; 4 isolates were susceptible to piperacillin and 3 to amikacin.

**Figure 1:**
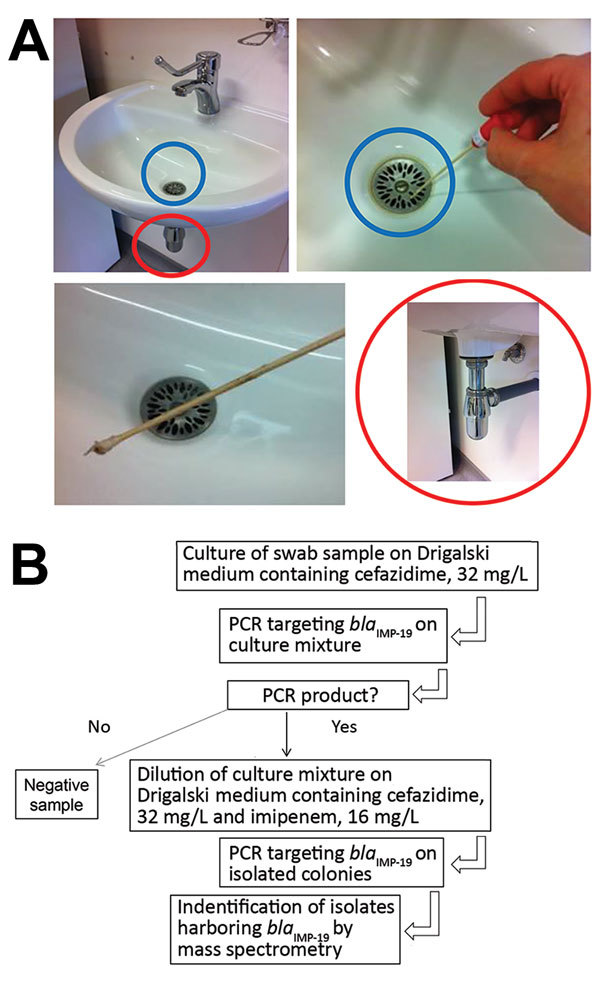
Environmental testing for IMP-19 producers in investigation of nosocomial infections linked to contaminated sinks, France. A) Sink (blue circles), swab specimen of sink, and drain (red circles) tested. B) Flowchart of testing procedure.

We conducted an environmental investigation in the hospital (Figure 1). More than 100 environmental samples were obtained when no patients were colonized (except for samples collected in room 32 a few hours after patient P7 had been transferred to an intensive care unit). Water samples were collected from different faucets (nursing room, medication preparation rooms, and rooms of some patients). First-catch lukewarm water (500 mL) was collected in sterile bottles containing 20 mg/L sodium thiosulfate and concentrated by filtration (0.45-μm membrane filters). All sinks and shower drains were sampled by rotating a cotton swab inserted through the drain. Toilets were sampled with swabs inserted under the rim of the toilet bowl.

We plated samples from filters (for water) and swab specimens (from sinks) on Drigalski medium containing ceftazidime (32 mg/L). When a positive culture was observed after 48 hours of incubation, a PCR specific for *bla*_IMP-19_ was performed for the culture mixture. For samples with a positive PCR result, we then plated a dilution of the culture mixture on Drigalski medium containing ceftazidime (32 mg/L) and imipenem (16 mg/L) to obtain isolated colonies, which we further subjected to identification and confirmation of IMP-19 production.

Many resistant organisms were detected in these environmental samples. We used mass spectrometry and *nrdA* gene sequencing to identify for *Achromobacter* isolates (*6*). *bla*_IMP-19_ and integrons were detected as described (*7**,**8*). We used pulsed-field gel electrophoresis after *Xba*I digestion to genotype *P. aeruginosa* isolates (*9*). Pulsotypes were compared by calculating the Dice correlation coefficient with DendroUPGMA software (http://genomes.urv.cat/UPGMA/).

**Figure 2:**
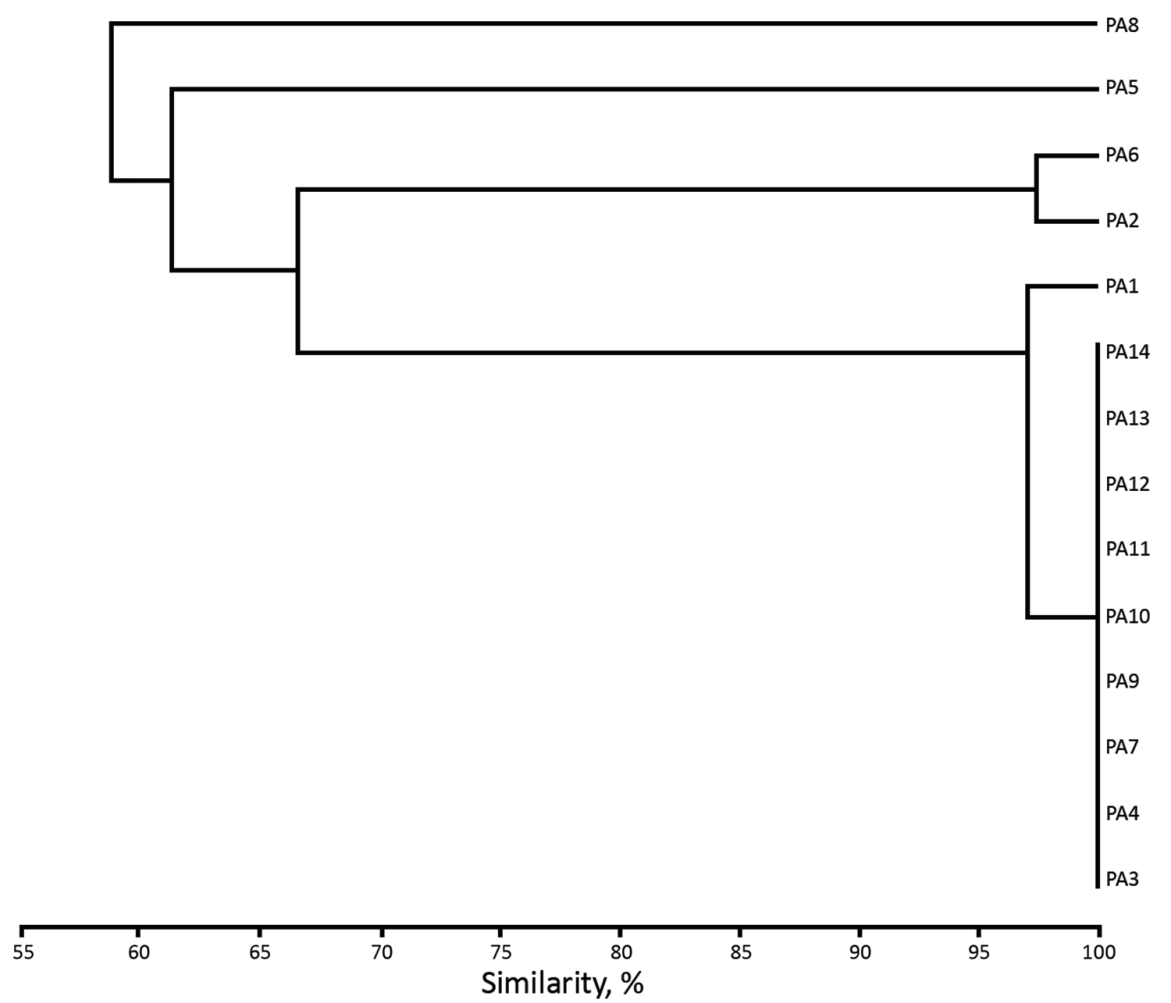
Unweighted pair group method with arithmetic mean cluster analysis of *XbaI*-generated pulsotypes constructed with Dice coefficients for the 7 clinical isolates and the 7 environmental isolates of IMP-19−producing *Pseudomonas aeruginosa* linked to contaminated sinks, France. Isolates are indicated on dendrogram branches. The Dice coefficient scale is indicated at the bottom of the dendrogram.

The 7 clinical isolates belonged to 3 distinct genotypes A, B, and C (Table; Figure 2). We detected environmental IMP-19–producing isolates belonging to 8 species of gram-negative nonfermenting bacilli: *P. aeruginosa*, *Achromobacter xylosoxidans*, *A. aegrifaciens*, *P. putida*, *Stenotrophomonas maltophilia*, *P. mendocina*, *Comamonas testosteroni*, and *Sphingomonas* sp. Of the 7 environmental isolates of *P. aeruginosa* we identified, 6 belonged to the same genotype as clinical isolates (genotype A).

**Table:** Characteristics of bacterial isolates from patients with nosocomial infections linked to contaminated sinks, France*

Isolate	Origin (date)	Site	PFGE profile	Integron
*Pseudomonas aeruginosa* PA1	Patient P1 (2009 Feb)	Urine	A	D
*P. aeruginosa* PA2	Patient P2 (2009 Apr)	Blood	B	A
*P. aeruginosa* PA3	Patient P3 (2009 Jun)	Throat	A	D
*P. aeruginosa* PA4	Patient P4 (2013 Oct)	Urine	A	D
*P. aeruginosa* PA5	Patient P5 (2015 Oct)	Central catheter	C	F
*P. aeruginosa* PA6	Patient P6 (2015 Dec)	Throat	B	A
*P. aeruginosa* PA7	Patient P7 (2016 Jul)	Blood	A	ND
*P. aeruginosa* PA8	Room 10 (protective unit)	Sink	Unrelated	G
*P. aeruginosa* PA9	Room 40	Shower drain	A	A
*P. aeruginosa* PA10	Room 40	Shower drain	A	E, F
*P. aeruginosa* PA11	Room 40	Sink	A	B, C
*P. aeruginosa* PA12	Room 46	Shower drain	A	D
*P. aeruginosa* PA13	Room 32	Sink	A	ND
*P. aeruginosa* PA14	Room 32	Toilet bowl	A	ND
*Achromobacter xylosoxidans*	Room 48	Sink	ND	F
*A. aegrifaciens*	Room 32	Toilet bowl	ND	E, F
*A. aegrifaciens*	Room 37	Shower drain	ND	E, F
*P. putida*	Room 32	Toilet bowl	ND	E, F
*P. putida*	Room 4	Shower drain	ND	E, F
*P. putida*	Room 64	Shower drain	ND	G
*P. putida*	Room 65	Toilet bowl	ND	C
*Sphingomonas *sp.	Room 12 (protective unit)	Sink	ND	ND
*Comamonas testosteroni*	Room 46	Sink	ND	ND
*P. mendocina*	Room 40	Shower drain	ND	E, F
*Stenotrophomonas maltophilia*	Room 44	Sink	ND	ND

*ND, not determined; PFGE, pulsed-field gel electrophoresis.

**Figure 3:**
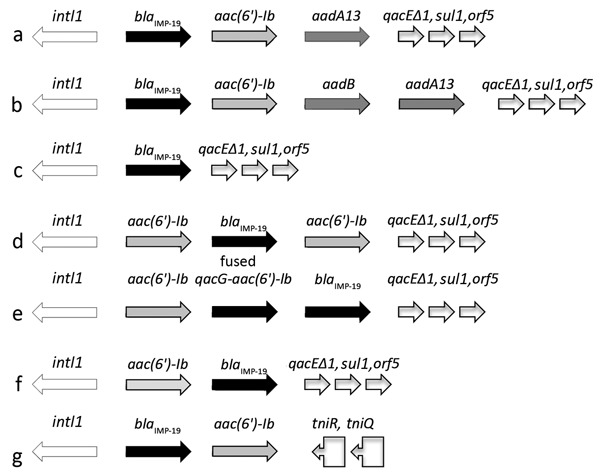
Diversity of integrons harboring *bla*_IMP-19_ isolated from patients with nosocomial infections linked to contaminated sinks, France. Arrows indicate direction of transcription. a–f, *suI*-type class 1 integrons; g, Tn*402*−like class 1 integron. *aac(6′)-Ib*, aminoglycoside 6′-N-acetyltransferase; *aadA13*, aminoglycoside adenyltransferase ANT(3′); *aadB*, aminoglycoside-2′′-O-nucleotidyltransferase; *bla*_IMP-19_, metallo-β-lactamase IMP-19; fused *qacG*, aminoglycoside 6′-N-acetyltransferase; *intl1*, class 1 integron integrase; *orf5*, open reading frame 5; *qacEΔ1*, multidrug exporter; *sul1,* dihydropteroate synthase; *tniQ*, transposition protein; *tniR*, resolvase.

The *bla*_IMP-19_ gene was located in various integrons, mainly on a *su*l-type class 1 integron and A *Tn402*-like class 1 integron. In these integrons, *bla*_IMP-19_ was associated with different gene cassettes, including *aac(6′)-Ib*, *aadA13*, *aadB*, or fused *qacG-aac(6′)-Ib* (Figure 3). Few isolates had several copies of *bla*_IMP-19_ located on integrons of different structures (Table).

## Conclusions

The incidence of MBL producers among imipenem-resistant *P. aeruginosa* in France is low compared with incidences for other countries (*2**,**10*). Reports of outbreaks are scarce and usually involve VIM producers (*11**,**12*). Therefore, detection of IMP-19 producers in our hospital was unusual. The long intervals without cases, the absence of any overlap between cases, and genotypic diversity of clinical isolates did not suggest a single common source of infection. These findings prompted us to conduct environmental investigations.

IMP-19 producers were detected in 9 of 15 rooms in the conventional unit and 2 of 9 rooms in the protective isolation unit. These producers were *P. aeruginosa* and a wide variety of gram-negative nonfermenting bacilli. Most of these producers have little clinical relevance, but they are silent reservoirs for dissemination of *bla*_IMP-19_ to major pathogens. The role of these environmental bacterial species in the spread of MBL suggested in previous studies (*13**,**14*) is confirmed by our findings.

The diversity of species found and genetic structures involved with *bla*_IMP-19_ indicated that the environmental contamination occurred a long time ago. One isolate of IMP-19–producing *Aeromonas caviae* was found in a patient in the same building in 2006 (*7*). This phenomenon is probably endemic to our hospital, in which sink drains are not accessible for removal of biofilms without complete dismantling (inappropriate sink design).

Transfer of pathogens from sinks to patients might occur in several ways. Water from faucets is directed straight into the drain, resulting in splashing that can lead to contamination of an area <1 m from the sink (*15*). Therefore, patients can be contaminated when they brush their teeth, wash their hands, or take a shower. Healthcare personnel are also at risk for hand contamination, which might lead to transfer of pathogens to patients during care.

All patients had >1 stay in rooms that were positive for IMP-19–producing organisms. After patient P7 died of sepsis, all drains in the ward were changed. However, this measure did not eradicate biofilms found in the plumbing system. Because the building tested was 16 years old, it has been decided to completely rebuild the ward in early 2017, paying special attention to water distribution and discharge systems to minimize hospital-acquired infections. In conclusion, our findings might help other hospitals to identify potential reservoirs of carbapenemase-producing bacteria and lead to implementation of rapid control measures to contain outbreaks.
